# Effect of xylo‐oligosaccharides (XOS) addition on technological and sensory attributes of cookies

**DOI:** 10.1002/fsn3.1802

**Published:** 2020-08-31

**Authors:** Réka Juhász, Péter Penksza, László Sipos

**Affiliations:** ^1^ Department of Dietetics and Nutrition Sciences Semmelweis University Budapest Hungary; ^2^ Department of Food Preservation Szent István University Budapest Hungary; ^3^ Department of Postharvest Sciences and Sensory Evaluation Faculty of Food Science Szent István University Budapest Hungary

**Keywords:** color measurement, nondigestible oligosaccharides (NDO), sensory panel performance, texture profile analyzer

## Abstract

Xylo‐oligosaccharides (XOS) are nondigestible oligosaccharides (NDO) which are recently authorized as novel food ingredients in European Union. Present study introduces the effect of XOS on baking quality of cookies. Color measurements proved that XOS enhance the caramelization during baking. Texture profile, geometry, and baking loss of cookies showed little changes due to XOS addition indicating that XOS are easy to incorporate into baking products. Based on sensory evaluation by expert panel, it was observed that XOS increased the “baked character” of the cookies as indicated by the increased caramel flavor, darker color, and crispier texture. XOS addition also increased the sweet taste and global taste intensity of cookies suggesting that in bakery products XOS evolve a flavor enhancer role. XOS proved to be a promising new alternative to increase dietary fiber content of cereal‐based cookies.

## Graphical abstract

Xylo‐oligosaccharides (XOS) were used for dietary fiber enrichment of cookies. Texture profile, colour and sensory attributes have been measured. It was concluded that XOS enhance brown colour, crispy texture and “baked character” of the cookies.

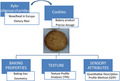

## 1 INTRODUCTION

There is a growing interest in high fiber diets due to their beneficial effects such as prevention of chronic diseases such as obesity, *diabetes mellitus*, colonic cancer, coronary heart disease, and caries (Pareyt, Goovaerts, Broekaert, & Delcour, 2011; Reynolds et al., 2019). Dietary fiber addition is usually cause positive health effect when used at high concentration; in cookies, it is typically 10‐30 g/100 g. At this level of use, dietary fiber usually causes lower quality such as decreased volume, decreased crispiness, or unpleasant dark color (Bilgicli, Sbenol, & Nur, 2007). High exposure to dietary fiber may also cause unpleasant symptoms such as gastrointestinal discomfort, laxative effect, or flatulence. Nondigestible oligosaccharides are reported to be effective prebiotics even at low concentration (Sako, Alonso, Domínguez, & Parajó, 1999).

Xylo‐oligosaccharides (XOS) are a new type of nondigestible oligosaccharides (NDO) and hence improve gut microecology including bacterial populations, and biochemical profiles therefore are claimed as prebiotics (Sako et al., 1999). Xylo‐oligosaccharides (XOS) are oligomers of two to ten *β*‐1,4‐linked xylose monomers and are hydrolysis products of xylan found in fruits, vegetables, bamboo, honey, milk, and in xylan‐rich lignocellulosic material obtained from agricultural, forestal, and industrial waste (Carvalho et al., 2015; Madhukumar & Muralikrishna, 2012; Vázquez et al., 1999). These oligosaccharides are stable at temperatures up to 100°C and over the wide pH range of 2.5–8.0 therefore in the gastric pH range as well (Courtin, Swennen, Verjans, & Delcour, 2009).

XOS are nondigestible, noncariogenic prebiotics. (Aachary, Gobinath, & Prapulla, 2011) They stimulate bacterial growth and fermentation and improve intestinal mineral absorption in addition to being antioxidants (Moure, Gullón, Domínguez, & Parajó, 2006). These compounds are also noncariogenic as the bacteria of human oral microflora are not able to metabolize them (Vázquez et al., 1999).

A minimum daily XOS intake is required to achieve health effects: Effective dose of XOS as prebiotics has been suggested to be as low as 1 g per day (Mäkeläinen, Juntunen, & Hasselwander, 2009). Regular XOS consumption in Asia confirming that positive impact of XOS on human health is related to a daily dose of 1–4 g/person. However, consuming more than 12 g XOS per day may result slight gastrointestinal effects in the case of sensitive consumers. These effects may be gastrointestinal discomfort, laxative effect, or flatulence are typical symptoms for high exposure to dietary fiber (Xiao, Ning, & Xu, 2012).

XOS are well known and widely used as a functional food ingredient or food supplement in Japan and China. In Japan, XOS are approved as food ingredients by Foods for Specified Health Uses (FOSHU) specifically foods that modify gastrointestinal conditions. In China, XOS have been commercialized since 2000 and have been used as a food supplement and as a functional compound in dairy products (Mäkeläinen et al., 2009). In Europe, XOS are a novel food ingredient and our research team has been working on having its use authorized based on suggestions of regulations (EC, 1997) No 258/97 and No 2015/2283 of the European Parliament. It has been accepted as novel food by the European Food Safety Authority (EFSA, 2018) and the European Commission as proved by Commission Implementing Regulation (EU) 2018/1648 of 29 October 2018. Our previous study introduced the first steps of this work testing the rheological properties of XOS in aqueous media (Penksza, Juhász, Szabó‐Nótin, & Sipos, 2020).

Since 1997, 32 product launches using XOS have been recorded by Mintel's global new products database (Mintel, 2008). The most typical products are dairy products, beverages, fruit juices, chewing gums, and healthcare products (dietary supplements). However, because of recent authorization of XOS limited information is available about its effect on quality of food products common in Europe such as bakery products, cookies, and breakfast cereals. Cookies are easy to dose because of well‐defined size and weight and are suitable for precisely control the XOS intake. Previous study with arabinoxylan oligosaccharides (AXOS) enriched cookies showed that baking quality of cookies depends on whether AXOS are used as a flour or as a sugar replacer (Pareyt et al., 2011).

Aim of present study was to investigate effect of xylo‐oligosaccharides addition on baking quality of cookies. Main objectives were to:

1. study changes of baking properties (baking loss, volume, color, texture),
2. ascertain the sensory profile of dietary fiber enriched modified cookies (flour or sugar was partly replaced by XOS),
3. evaluate the differences between available XOS products (powder and liquid forms, purity of 70 or 95%) when are used in cookies.

## 2 MATERIALS AND METHODS

### 2.1 Materials

Commercial flour (moisture level 11.17 g/100 g; water absorption 63.3%) was from GoodMills (Komárom, Hungary), sucrose from Magyar Cukor Zrt. (Budapest, Hungary), margarine from Unilever (Budapest, Hungary), and sodium bicarbonate from Szilasfood (Kistarcsa, Hungary). Flour moisture was determined with AACC‐approved method 44‐19 (AACC, 1983), and the flour water absorption was determined by Farinograph (Brabender). Three types of XOS were from Longlive Shandong (China). 95P (powder, contains 95% XOS), 70 L (syrup, contains 70% XOS), and 70P (powder, contains 70% XOS) were used in the experiment.

### 2.2 Sample preparation

Cookie doughs were prepared according to the AACC‐approved Method 10‐50D (AACC, 1980). Three kinds of recipes were used in our experiments. The ingredients and formulas of the three kinds of recipes are introduced in Table  1. The control sample contained the ingredients according to the standard. XOS were used to replace a part of flour (sample code: F70, F70L, F95P) and a part of sugar (sample code: S70P; S70L; S95P). XOS were used in 1.4% concentration level because of nutritional considerations.

**TABLE 1:** Recipe of cookies with or without xylo‐oligosaccharides (XOS) addition

	Control	F70P	S70P	F70L	S70L	F95P	S95P
Wheat flour (g)	225.0	216.25	225.0	216.25	225.0	218.68	225.0
Sugar (g)	130.0	130.0	121.25	121.25	130.0	130.0	121.25
Fiber (XOS) (g)	0	8.75	8.75	8.75	8.75	6.32	6.32
Margarine (g)	64.0	64.0	64.0	64.0	64.0	64.0	64.0
Salt (g)	2.1	2.1	2.1	2.1	2.1	2.1	2.1
Sodium bicarbonate (g)	2.5	2.5	2.5	2.5	2.5	2.5	2.5
Deionized water (ml)	16.0	16.0	16.0	16.0	16.0	16.0	16.0
Glucose syrup (ml)	33.0	33.0	33.0	33.0	33.0	33.0	33.0

Abbreviations: 70L, xylo‐oligosaccharides in liquid form with 70% XOS content; 70P, xylo‐oligosaccharides in powder form with 70% XOS content; 95P, xylo‐oligosaccharides in powder form with 95% XOS content. Glucose syrup (ml) (5.93g/100ml); F, flour replacement; S, sugar replacement.

Dough was prepared with one‐stage mixing, in which margarine and flour were homogenized first during 5 min. Then, the other components were added and mixing was continued for 10 min. Dough was laminated (7 mm thickness), then cut with a circular cookie cutter (inner diameter 50 mm), and baked in an electrically heated rotary oven (Gierre, Milano, Italy) for 10 min at 205°C.

### 2.3 Methods

#### 2.3.1 Geometry

After cooling to room temperature, cookies were weighed and their dimensions (height, diameter in cm) measured, and from these parameters, the volume was calculated: (V = r^2^*π*h). Baking loss (Bl) was determined using the following equation:

Bl* = *((w_1_‐w_2_)/w_1_)×100

where

*w*_1_ is the weight of cookie prior to baking,

*w*_2_ is the weight of cookie after baking.

#### 2.3.2 Color

Color of the surface of the baked cookies was measured with 9 parallels using a Konica Minolta CR400 chromameter. Results were expressed as CIE 1976 L*, a*, and b* values. L* is a measure of the brightness from black (0) to white (100), while a* describes the red‐green color (a*> 0 indicates redness, a* < 0 indicates greenness), and b* describes yellow‐blue color (b*> 0 indicates yellowness, b* < 0 indicates blueness). To determine the total color difference between two samples using all the three coordinates, the following formula was used (CIE, 1976): $\Delta E_{a,b}^{*}=\sqrt{{\left(L_{2}‐L_{1}\right)}^{2}+{\left(a_{2}‐a_{1}\right)}^{2}+{\left(b_{2}‐b_{1}\right)}^{2}}$ (CIE, 2004).

#### 2.3.3 Texture profile analyses

Texture of cookies was characterized using a Brookfield LFRA Texture Analyzer (LFRA 4500 Texture Analyser; Brookfield, Middleboro, USA) and equipped with the probe‐type TA43 (metal needle). Nine cookies were selected, and the measurement was done on their center. The data were recorded, and the analysis of the texture profile was performed by using TexturePro Lite v1.1 Build 4 software. The dimensions of certain texture parameters are given in the default form provided by the software. The test parameters were as follows: total cycles 2, test speed 2 mm*/s*, and target value 4 mm (distance reached by the test piece in the sample). Based on the texture profile load (*g*) in function of time (*s*), hardness (*g*) (maximum deformation force during the first mastication cycle), and adhesive force (*g·s*) (force required to pull the compressing plunger away from the sample), cohesiveness (*‐*), springiness (*mm*), gumminess (*g*), and chewiness (*g·mm*) were determined.

#### 2.3.4 Sensory analysis

Sensory evaluations were conducted at Szent István University, Sensory Evaluation Laboratory, which meets standard requirements (ISO 8589:^2007^). The panel consisted of 12 trained assessors (six females and six male, at the age of 20 to 28) with necessary knowledge and experience in sensory descriptive analysis, which included techniques and practice in attribute identification and terminology development. These trained individuals went through training which met the standard requirements (ISO 8586:ISO, 2012a, ISO, 2012b). The trained sensory panel tested a wide range of bakery products, including a number of commercially available biscuit sensory profiles, so panelists were highly skilled in the sensory profiling of these products.

The trained panel sensory tests were carried out using quantitative descriptive profile (QDP) Method (ISO 13299:^2016^). The trained panel evaluated the cookies using a scale between 0 and 100 for each. The panelists had analyzed 27 attributes, which involved appearance—brightness, color hue, shape, high, diameter, and homogeneity of surface; texture—hardness, chewiness, cohesiveness, crispiness, and mouthcoating; odor—global odor intensity, flour odor intensity, sweet odor intensity, caramel odor intensity, margarine odor intensity, baking soda odor intensity, and salt odor intensity; taste/flavor—global taste intensity, flour taste intensity, sweet taste intensity, caramel taste intensity, margarine taste intensity, baking soda flavor intensity, salt flavor intensity, off‐taste intensity, after‐taste intensity—properties. In order to prevent sensory fatigue, there was a half‐hour break between appearance/texture attributes, odor attributes, and taste attributes. Each sensory property and values of the control sample were determined by consensus. Seven cookie samples were evaluated by panel. Tests were conducted using two replicates to ensure data reliability. The sessions were conducted in 4 different days to achieve proper repetitions; hence, one control samples and three other samples were tested each day. The sessions were held during morning (between 10 a.m. and 12 a.m.) because in this time the human senses are the most sensitive (ISO 6658:^2017^).

#### 2.3.5 Statistical methods

The sensory attributes of the cookies were evaluated separately. The mean values were compared by 2‐way analysis of variance (ANOVA) when evaluating the sensory attributes of the products (*α* = 0.05). Pair comparison was done by Tukey HSD post hoc test. These tests were carried out using XL‐STAT software created by Addinsoft.

The performance monitoring of the panel was carried out according to the workflow of the PanelCheck ver 1.4.2 software using one‐way and multiway statistical methods: detecting the nonsignificant product effects (2‐way ANOVA, *α* = 0.05); identifying panelists who differ from the rest of the panel (Tucker‐1 plot, Manhattan plot); and analyzing the discriminating ability of the panelists (F plots, *MSE* plots, *p* ∗ *MSE* plots) (ISO 11132:ISO, 2012a, ISO, 2012b; Naes et al. 2010; Tomic et al. 2010).

The performance of the trained sensory panel was analyzed by mixed assessor model‐control of assessor performance (MAM‐CAP) table method for testing discrimination, agreement, repeatability, and scaling at panel. The MAM‐CAP table was created in R‐project ver. 3.6.1 with MAM‐CAP‐package (Peltier, Brockhoff, Visalli, & Schlich, 2013; R‐project, 2019).

## 3 RESULTS AND DISCUSSION

### 3.1 Results of the panel performance monitoring

After the 2‐way ANOVA analysis, all sensory attributes were significant (*p* < .05), so there were no attributes that not used during the further data analysis. The 2‐way ANOVA model was the following: Attribute = Sample +Assessor + Sample∗Assessor. According to the Tucker‐1 analysis and Manhattan plots, the panel has a good performance too and gave similar responses to the same sensory attributes. The panel members located very close to each other on the outer ellipse except one attribute (after‐taste intensity). Assessors reached high explained variance using just the first 2 or 3 PC’s. It can be claimed that panel members agreed well between the evaluated attributes. When analyzing, *F* plots in the assessors could not discriminate so well the products according to the attributes of baking soda odor/taste intensity, salt odor/taste intensity, off‐taste intensity, and after‐taste intensity. The products in these properties were very similar. The *MSE* values show how similar are the given values of an assessor to the same stimulus, which means how consistent the assessor is. During our study, most of the *MSE* values were close to the zero. These values show very good repeatability so the repeatability of the panel is good.

The MAM‐CAP table presents the panel performance. The MAM‐CAP table shows that this panel is globally well trained. All *F*‐Prod and *F*‐Disag values proved to be discriminant (*F*‐Prod *p* < .05, *F*‐Disag *p* > .05), and all attributes can be used in further analysis. With the exception of off‐taste and after‐taste, the *F*‐Scal value was adequate for all sensory characteristics (*F*‐Scal *p* > .05). The root‐mean‐square error (RMSE) gives an indication of panel repeatability: All sensory attributes were very good (RMSE ≤ 3.19) (Table 2.).

**TABLE 2:** Panel performance MAM‐CAP table

Attribute	Mean	*F*‐Prod	*F*‐Scal	*F*‐Disag	RMSE
Color hue	63.49	1845.01	0.11	1.36	2.00
Brightness	71.48	952.25	0.51	1.29	2.27
Cohesiveness	61.65	601.95	1.38	1.12	2.47
Diameter	51.73	521.2	1.41	1.47	1.94
Margarine odor intensity	12.92	511.75	0.30	1.36	1.41
Margarine flavor intensity	13.04	390.03	0.20	1.29	1.68
Baking soda flavor intensity	13.04	390.03	0.20	1.29	1.68
Caramel odor intensity	17.11	225.02	0.38	1.36	1.07
Global odor intensity	78.49	208.62	0.46	1.49	2.39
Sweet odor intensity	42.04	186.11	0.26	1.11	2.32
Crispiness	5.83	158.38	1.43	1.36	1.02
High	64.48	146.56	1.11	1.29	1.77
Homogeneity of surface	63.27	143.46	0.13	1.36	2.00
Shape	74.89	140.85	0.11	1.11	2.08
Flour odor intensity	36.05	124.81	0.29	1.49	2.86
caramel flavor intensity	14.45	104.13	0.06	1.47	2.15
Sweet taste intensity	78.03	103.97	1.49	1.36	2.31
Global taste intensity	77.45	84.22	0.10	1.12	2.12
Flour flavor intensity	50.89	55.67	0.65	0.89	3.19
Chewiness	13.35	49.64	0.57	1.09	1.52
Hardness	12.54	41.02	1.26	1.29	1.55
Mouthcoating	8.27	10.03	0.21	1.47	1.46
Salt taste intensity	3.71	10.02	0.60	1.36	1.36
Off‐taste intensity	0.05	5.30	7.54	0.42	0.31
After‐taste intensity	0.10	4.30	14.52	0.33	0.61
Baking soda odor intensity	3.55	2.30	0.26	1.36	1.36
Salt odor intensity	2.54	2.29	0.33	1.29	1.62

Note

The first column contains the mean of the panel for each attribute. The four following columns are, respectively: *F* statistics of discrimination (*F‐*Prod), scaling heterogeneity (*F‐*Scal) and disagreement (*F‐*Disag), and repeatability (root‐mean‐squares of error, RMSE). Attributes are sorted from the most discriminative to the less discriminative (*F‐*Prod) (panel limit = 0.05).

### 3.2 Baking properties

#### 3.2.1 Color

Color has primarily importance in acceptability of cookies by consumers and an important parameter in determination of baking quality. Color of cookies was strongly influenced by XOS addition as proved by both the sensory evaluation and instrumental measurement (Table 3).

**TABLE 3:** Color of cookies with or without xylo‐oligosaccharides (XOS) addition

	Control	F70L	F70P	F95P	S70L	S70P	S95P
	X ± *SD*	X ± *SD*	X ± *SD*	X ± *SD*	X ± *SD*	X ± *SD*	X ± *SD*
Measured							
L*	73.43 ± 1.87^c^	65.74 ± 2.84^ab^	67.21 ± 2.34^ab^	69.72 ± 3.26^bc^	64.58 ± 1.16^a^	67.12 ± 2.55^ab^	68.84 ± 2.51^bc^
a*	1.45 ± 1.96^a^	5.77 ± 1.40^bc^	5.78 ± 1.76^bc^	4.66 ± 2.50^ab^	7.88 ± 0.65^c^	6.25 ± 1.84^bc^	4.94 ± 1.49^ab^
b*	31.41 ± 1.78^a^	35.53 ± 1.12a^b^	37.63 ± 0.74^c^	36.59 ± 0.78^bc^	37.59 ± 0.72^c^	37.11 ± 1.03^bc^	36.22 ± 1.11^bc^
ΔE*_ab_	‐	9.20 (huge)	9.12 (huge)	6.56 (huge)	13.45 (huge)	16.25 (huge)	11.24 (huge)
Perceived							
Brightness	30.0^d^	70.7 ± 2.53^c^	68.4 ± 2.73^c^	78.8 ± 2.98^b^	79.2 ± 3.28^b^	86.4 ± 3.37^a^	87.0 ± 3.14^b^
Color hue	10.0^e^	60.1 ± 3.56^d^	61.7 ± 2.14^d^	72.0 ± 2.94^c^	79.0 ± 2.09^b^	81.6 ± 2.7^a^	80.1 ± 0.6^ab^

Note

The ΔE*_ab_ color differences with the control sample. Critical values: ΔE*_ab_ < 1.5: not perceptible; 1.5 < ΔE_ab_*<3.0: perceptible; 3.0 < ΔE_ab_* <6.0:well perceptible; 6.0 < ΔE_ab_*: huge.

Abbreviations: 70L, xylo‐oligosaccharides in liquid form with 70% XOS content; 70P, xylo‐oligosaccharides in powder form with 70% XOS content; 95P, xylo‐oligosaccharides in powder form with 95% XOS content superscript letters (a,b,c) indication of homogeneous and heterogeneous groups tested by Tukey HSD test at 95% confidence level; F, flour replacement; S, sugar replacement.

Brightness and color hue increased highly compared to control due to XOS addition according to panelists. Lightness (L*) decreased when XOS 70P or 70L was added to cookies, but did not show significant change when 95P was used. Red/green color (a*) had positive value (1.45–7.88) in the case of cookies indicating their red color, and it increased significantly in the case of every type of XOS except for 95P. Yellow/blue color (b*) had positive value (31.41–37.63) in the case of cookies indicating their yellow color, and it increased significantly in the case of every type of XOS. Color difference (ΔE*_ab_) between control and XOS added cookies was above 6.0 indicating huge difference.

Cookies with XOS addition showed more intensive browning during baking than control. It was indicated by the simultaneous decrease of L* and increase of a* and b* values. The biggest effect was observed when XOS 70P was used for sugar replacement and the smallest when XOS95P was used for flour replacement as indicated by the ΔE*_ab_ values (16.25 and 6.56, respectively).

Browning of cookies during baking was caused by Maillard reaction and caramelization. Cerny (2007) reported that xylose, the monomer of XOS, has a stronger browning effect than hexoses or sucrose. XOS 70P is produced with maltodextrin as excipient, which can also be involved in Maillard reaction and so contribute to the formulation of melanoidines responsible for the brown color (Belitz, Grosch, Schieberle, and (2009).

#### 3.2.2 Geometry and texture

Geometry is an important parameter in baking quality of cookies. In general, increased volume of baking products is associated with their improved quality (Hoseney & Rogers, 1994; Pareyt et al., 2011). Volume of cookie products is influenced by flour quality, gluten content, and quality of flour, water binding capacity of dough. The higher cookie is the better, and when volume is increased by diameter, it indicates the spread of the cookie. Baking loss refers to the moisture loss during baking: The higher water binding capacity of dough is related to lower baking loss.

XOS addition slightly affected the geometry of cookies (Table 4).

**TABLE 4:** Geometry and outlook of cookies with or without xylo‐oligosaccharide (XOS) addition

	Control	F70L	F70P	F95P	S70L	S70P	S95P
	X ± *SD*	X ± *SD*	X ± *SD*	X ± *SD*	X ± *SD*	X ± *SD*	X ± *SD*
Measured							
Baking loss (g)	2.29 ± 0.19^a^	2.51 ± 0.18^b^	2.60 ± 0.34^b^	2.47 ± 0.16a^b^	2.08 ± 0.20^b^	2.19 ± 1.28^ab^	2.52 ± 0.1^b^
Moisture (g/100g)	8.02 ± 0.06^a^	8.20 ± 0.09^a^	7.88 ± 0.56^a^	8.90 ± 0.25^a^	8.20 ± 0.10^a^	8.74 ± 0.56^a^	8.57 ± 0.25^a^
Diameter (cm)	5.26 ± 0.22^a^	5.56 ± 0.23^b^	5.43 ± 0.26^ab^	5.44 ± 0.32^ab^	5.43 ± 0.11a^b^	5.34 ± 0.14a^b^	5.39 ± 0.17a^b^
Height (cm)	2.38 ± 0.24^c^	2.19 ± 0.18^bc^	2.06 ± 0.15^ab^	2.04 ± 0.19^ab^	1.98 ± 0.10^a^	2.25 ± 0.19^bc^	2.16 ± 0.19^abc^
Volume (cm^3^)	43.81 ± 4.00^a^	53.33 ± 7.03^c^	47.66 ± 5.28^abc^	47.62 ± 6.67^abc^	45.72 ± 2.36^ab^	50.33 ± 4.76^bc^	49.20 ± 5.48a^bc^
Perceived							
Shape	90.0^a^	74.6 ± 2.48^b^	71.0 ± 2.23^d^	71.4 ± 2.86^cd^	73.2 ± 2.91^bc^	71.1 ± 1.62^d^	72.9 ± 2.52^bcd^
High	60.0^d^	70.3 ± 1.85^b^	54.7 ± 2.88^e^	68.5 ± 3.04^b^	64.0 ± 3.15^c^	60.1 ± 1.53^d^	73.8 ± 1.99^a^
Diameter	30.0^d^	50.2 ± 2.12^c^	49.0 ± 1.94^c^	50.0 ± 1.64^c^	62.1 ± 3.00^a^	59.5 ± 3.27^b^	61.3 ± 2.29^ab^
Homogeneity of surface	80.0^a^	61.4 ± 2.22^b^	58.0 ± 2.68^d^	61.2 ± 2.91^bc^	61.5 ± 2.40^bc^	60.2 ± 3.12^c^	60.5 ± 1.87^c^

Abbreviations: 70L, xylo‐oligosaccharides in liquid form with 70% XOS content; 70P, xylo‐oligosaccharides in powder form with 70% XOS content; 95P, xylo‐oligosaccharides in powder form with 95% XOS content superscript letters (a,b,c) indication of homogeneous and heterogeneous groups tested by Tukey HSD test at 95% confidence level; F, flour replacement; S, sugar replacement.

Diameter of cookies slightly increased because of addition any type of XOS measured by both analytical and sensory methods. It was 5.26 cm in the case of control and 5.34–5.56 cm in the case of XOS containing cookies. Similar changes were observed when flour or sugar was replaced by XOS. Height of cookies slightly decreased when XOS was added though it was scarcely perceptible by the panelists. Volume slightly increased (F70L, S70P, S95P) or not changed significantly (F70P, F95P, S70L) due to XOS addition.

Shape sensory attribute scores decreased significantly in the case of any type of XOS indicating that cookies became more uneven and irregular due to XOS addition. Homogeneity of surface got significantly less scores in the case of XOS addition because more holes, pores, and cracks occurred.

Moisture content of cookies did not change significantly because of XOS. Baking loss increased when flour was replaced by XOS. That may be due to dilution of flour gluten proteins resulting decrease of water binding of the flour as suggested by Pareyt et al. (2011) in the case of using arabinoxylan oligosaccharides (AXOS).

XOS addition at low concentration (1.4 w/w%) influenced geometry and outlook of cookies suggesting that xylo‐oligosaccharides change the water binding and water holding capacity of dough. Rheological properties of xylo‐oligosaccharides were reported to be different from sucrose by the authors (Penksza et al., 2019). XOS has higher viscosity in aqueous solution than sucrose, especially at room temperature (at which dough formation was performed) because of higher water binding capacity. Thus, XOS may interact with starch in a competitive manner for water or modify protein–starch–water interaction during gluten network formation resulting in a more uneven cookie shape and more heterogeneous surface.

**FIGURE 1:**
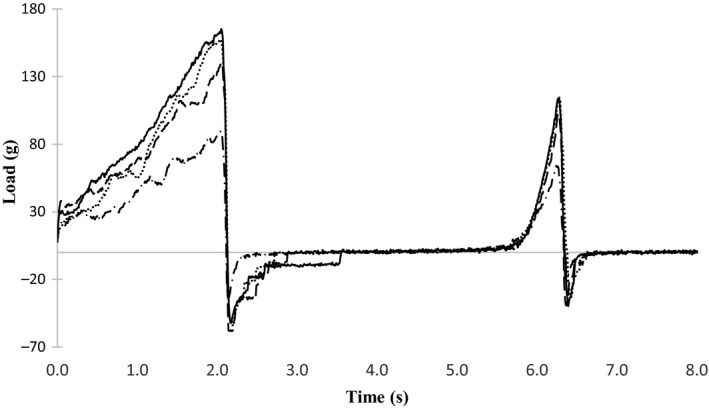
Texture profile of cookies in that flour is replaced by xylo‐oligosaccharides. 70L, xylo‐oligosaccharides in liquid form with 70% XOS content; 70P, xylo‐oligosaccharides in powder form with 70% XOS content; 95P, xylo‐oligosaccharides in powder form with 95% XOS content; F, flour replacement. ··········, Control; – – –, F70P; –––––, F70L; –·–·–, F95P

**FIGURE 2:**
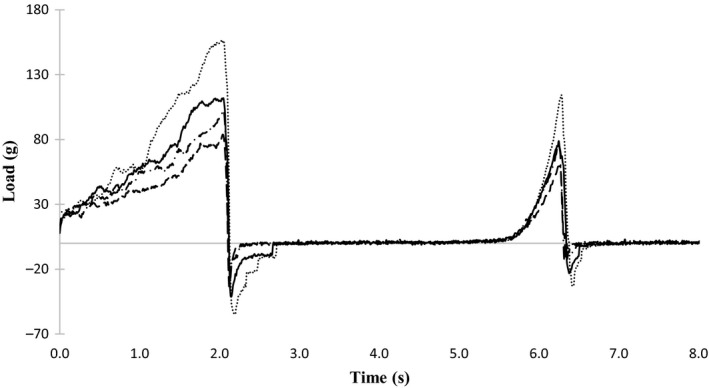
Texture profile of cookies in that sugar is replaced by xylo‐oligosaccharides. 70L, xylo‐oligosaccharides in liquid form with 70% XOS content; 70P, xylo‐oligosaccharides in powder form with 70% XOS content; 95P, xylo‐oligosaccharides in powder form with 95% XOS content; S, sugar replacement. ··········, Control; – – –, S70P; –––––, S70L; –·–·–, S95P

XOS addition influenced the texture of cookies (Figures 1 and 2.), and it was more intensively noticed by the sensory panelists than by the texture analyzer equipment. Differences in sensitivity may caused by the heterogeneity of the cookies causing great standard deviation of texture parameters determined by equipment.

In the case of S70L, F70L, and S95P, neither of the texture parameters have been changed by XOS addition (Table 5). S70P affected the most of the parameters as decreased hardness, gumminess, and chewiness. F95P decreased hardness and adhesive force, while F70P decreased chewiness. Hardness did not change or slightly increased, and cohesiveness decreased as according to the panelists. Crispiness and chewiness increased. It means that cookies became more crumbly in the presence of XOS. Mouthcoating increased due to XOS addition. It can be caused by presence of fat or by increased amount of bound water in cookies.

**TABLE 5:** Texture parameters of the cookies with or without xylo‐oligosaccharide (XOS) addition

	Control	F70L	F70P	F95P	S70L	S70P	S95P
	X ± *SD*	X ± *SD*	X ± *SD*	X ± *SD*	X ± *SD*	X ± *SD*	X ± *SD*
Measured							
Hardness (g)	163 ± 36.11^b^	168 ± 48.34^b^	146 ± 49.85^ab^	98 ± 17.94^a^	128 ± 26.74^ab^	90 ± 21.35^a^	117 ± 30.88^ab^
Cohesiveness	0.21 ± 0.03^a^	0.18 ± 0.03^a^	0.19 ± 0.05^a^	0.23 ± 0.04^a^	0.21 ± 0.05^a^	0.21 ± 0.01^a^	0.21 ± 0.02^a^
Gumminess	33 ± 8.06^b^	30 ± 6.66^b^	25 ± 5.19^ab^	22 ± 4.76^ab^	26 ± 4.57^ab^	18 ± 4.47^a^	24 ± 5.31^ab^
Chewiness	33 ± 12.3^b^	23 ± 5.78^ab^	19 ± 4.19^a^	21 ± 3.87^ab^	24 ± 4.16^ab^	17 ± 5.95^a^	24 ± 6.35^ab^
Adhesive force (g)	66 ± 17.48^a^	65 ± 13.32^a^	66 ± 17.11^ab^	42 ± 8.98^b^	54 ± 8.74^ab^	44 ± 8.25^ab^	51 ± 13.79^ab^
Springiness (mm)	0.98 ± 0.16^a^	0.77 ± 0.09^a^	0.75 ± 0.10^a^	0.96 ± 0.14^a^	0.94 ± 0.10^a^	0.90 ± 0.14^a^	0.97 ± 0.12^a^
Perceived							
Hardness	10.0^d^	10.5 ± 0.98^cd^	11.2 ± 1.93^cd^	13.5 ± 1.96^b^	16.4 ± 2.10^a^	14.4 ± 1.47^b^	11.8 ± 2.25^c^
Chewiness	10.0^b^	14.4 ± 1.4^a^	10.1 ± 1.08^b^	15.6 ± 2.50^a^	14.5 ± 1.84^a^	14.4 ± 1.61^a^	14.4 ± 1.52^a^
Cohesiveness	80.0^a^	47.3 ± 2.97^f^	52.5 ± 3.61^e^	48.4 ± 2.70^f^	76.6 ± 3.23^b^	62.0 ± 2.39^d^	64.7 ± 0.95^c^
Crispiness	0.0^d^	5.37 ± 0.87^c^	4.88 ± 0.61^c^	5.04 ± 0.20^c^	11.1 ± 2.41^a^	5.04 ± 1.23^c^	9.42 ± 1.74^b^
Mouthcoating	5.0^c^	7.79 ± 2.55^b^	9.50 ± 1.29^a^	8.79 ± 2.06^ab^	9.17 ± 1.90^ab^	8.42 ± 2.04^ab^	9.21 ± 1.69^ab^

Abbreviations: 70L, xylo‐oligosaccharides in liquid form with 70% XOS content; 70P, xylo‐oligosaccharides in powder form with 70% XOS content; 95P, xylo‐oligosaccharides in powder form with 95% XOS content superscript letters (a,b,c) indication of homogeneous and heterogeneous groups tested by Tukey HSD test at 95% confidence level; F, flour replacement; S, sugar replacement.

#### 3.2.3 Sensory attributes

Effect of XOS on sensory attributes of cookies has not been described previously; therefore, it was evaluated in detail (Table 6).

**TABLE 6:** Sensory attributes of cookies with or without xylo‐oligosaccharide (XOS) addition

	control	F70L	F70P	F95P	S70L	S70P	S95P
		X ± *SD*	X ± *SD*	X ± *SD*	X ± *SD*	X ± *SD*	X ± *SD*
Global odor intensity	90.0^a^	62.9 ± 2.45^e^	69.5 ± 3.06^d^	79.2 ± 3.30^c^	82.1 ± 3.24^bc^	84.1 ± 2.46^b^	81.6 ± 2.83^c^
Flour odor intensity	50.0^a^	31.0 ± 3.8^d^	38.1 ± 2.79^c^	40.9 ± 2.27^b^	26.6 ± 3.78^e^	30.2 ± 1.43^d^	38.8 ± 4.54^c^
Sweet odor intensity	30.0^e^	43.4 ± 2.24^b^	48.0 ± 2.51^a^	38.0 ± 2.81^d^	47.9 ± 2.32^a^	40.8 ± 2.52^c^	48.7 ± 2.85^a^
Caramel odor intensity	5.0^e^	21.46 ± 2.50^b^	19.13 ± 1.73^c^	10.3 ± 1.33^d^	20.3 ± 2.81^bc^	19.92 ± 0.41^bc^	23.96 ± 2.46^a^
Margarine odor intensity	30.0^a^	12.21 ± 2.21^b^	10.0 ± 1.72^c^	10.0 ± 1.27^c^	9.42 ± 1.02^c^	9.38 ± 1.93^c^	9.38 ± 1.50^c^
Baking soda odor intensity	3.0^b^	2.67 ± 2.28^b^	2.88 ± 2.15^b^	3.96 ± 2.26^ab^	3.71 ± 2.20^ab^	4.75 ± 0.53^a^	3.88 ± 1.87^ab^
Salt odor intensity	3.0^ab^	2.92 ± 2.24^ab^	2.88 ± 2.44^ab^	3.29 ± 2.45^b^	2.37 ± 1.97^ab^	1.83 ± 2.14^ab^	1.50 ± 1.77^b^
Global taste intensity	65.0^c^	78.0 ± 2.27^b^	80.9 ± 1.77^a^	78.3 ± 2.68^b^	81.1 ± 1.74^a^	81.6 ± 3.73^a^	77.3 ± 2.83^b^
Flour flavor intensity	60.0^a^	47.71 ± 3.50^cd^	45.3 ± 4.63^d^	50.13 ± 2.94^bc^	50.21 ± 2.73^bc^	51.9 ± 2.54^b^	51.1 ± 2.93^b^
Sweet taste intensity	70.0^f^	78.13 ± 3.87^cd^	77.0 ± 2.84^b^	72.9 ± 2.21^e^	88.4 ± 2.96^a^	81.3 ± 2.88^b^	80.4 ± 1.71^bc^
Caramel flavor intensity	3.0^d^	12.6 ± 2.72^c^	17.9 ± 2.65^a^	16.0 ± 1.71^b^	15.5 ± 2.62^b^	18.0 ± 2.40^a^	18.1 ± 2.42^a^
Margarine flavor intensity	30.0^a^	7.71 ± 2.54^e^	9.63 ± 1.13^d^	9.21 ± 1.50^d^	10.1 ± 1.12^cd^	11.4 ± 2.02^c^	13.2 ± 2.11^b^
Baking soda flavor intensity	2.0^c^	4.67 ± 1.05^b^	4.79 ± 0.41^ab^	5.50 ± 1.84^a^	4.75 ± 0.68^ab^	5.00 ± 0.51^ab^	5.00 ± 0.29^ab^
Salt taste intensity	2.0^c^	4.25 ± 1.42^ab^	4.29 ± 1.27^ab^	3.08 ± 2.15^bc^	3.83 ± 1.79^b^	5.50 ± 1.44^a^	3.00 ± 1.89^bc^
Off‐taste intensity	0.0^a^	0.25 ± 0.68^a^	0.00 ± 0.00^a^	0.00 ± 0.00^a^	0.00 ± 0.00^a^	0.00 ± 0.00^a^	0.08 ± 0.41^a^
After‐taste intensity	0.0^a^	0.08 ± 0.41^a^	0.08 ± 0.41^a^	0.00 ± 0.00^a^	0.42 ± 1.41^a^	0.00 ± 0.00^a^	0.08 ± 0.41^a^

Abbreviations: 70L, xylo‐oligosaccharides in liquid form with 70% XOS content; 70P, xylo‐oligosaccharides in powder form with 70% XOS content; 95P, xylo‐oligosaccharides in powder form with 95% XOS content superscript letters (a,b,c) indication of homogeneous and heterogeneous groups tested by Tukey HSD test at 95% confidence level; F, flour replacement; S, sugar replacement.

The presence of XOS did not cause any off‐taste or after‐taste in cookies. Baking soda flavor and salt taste were not perceptible by panelists neither in control nor in XOS containing cookies. Addition of XOS to cookies increased their global taste intensity (65.0 for control and 77.3–81.6 for XOS containing samples), sweet taste intensity (70.0 for control and 72.9–88.4 for XOS containing samples), and caramel flavor intensity (3.0 for control and 12.6–18.1 for XOS containing samples). Sweet taste is caused by the XOS as it is reported to have a sweeting powder 0.3–0.6 relative to sucrose (Vázquez et al., 1999), while caramel aroma compounds are produced by Maillard reaction (Belitz et al., 2009).

XOS addition decreased flour and margarine flavor intensity. Flour flavor was intensively perceived by panelists in the case of control cookie (score: 60 points), and it was still strong when XOS was added (45.3–51.9 points). Margarine flavor was perceived to a lesser extent, though its intensity showed a marked decrease because of the presence of XOS. (30 for control and 7.71–13.2 points for XOS containing samples). Flour and margarine flavor contributes to the character of the dough and decreases during baking the cookies. Increase of sweet taste and caramel flavor and decrease of flour and margarine flavor altogether indicate that XOS addition increased the “baked character” of cookies—not only by color but by taste. Xylose, the monomer of xylo‐oligosaccharides, is used in food industry to promote forming pleasant brown color and “baked flavor” of food products such as ready‐to‐eat meals and meats. Similar tendencies have been observed in the case of odor as in the case of taste with one exception: Global odor intensity decreased after XOS addition. Baking soda and salt odor intensity were scarcely perceptible. Sweet odor intensity increased, and caramel odor intensity intensively increased in the presence of XOS. Flour and margarine odor intensity decreased because of XOS addition. These results confirm the taste results indicating that XOS addition increased the “baked character” of cookies.

## 4 CONCLUSION

XOS are recently accepted in the European Union as novel food ingredient (Commission Implementing Regulation (EU) 2018/1648 of October 29, 2018); therefore, limited information is available about its effect on quality of food products common in Europe. Color measurement results proved that cookies browned more intensively in the presence of XOS. Based on sensory evaluation by expert panel, it was observed that XOS increased the “baked character” of the cookies as indicated by the increased caramel flavor, darker color, and crispier texture. XOS addition also increased the sweet taste and global taste intensity of cookies suggesting that in bakery products XOS play a flavor enhancer role. Further investigation needed to ascertain consumers’ preference (preference mapping) and regarding the changes occurred in presence of XOS.

## CONFLICT OF INTEREST

The authors declare that they do not have any conflict of interest.
